# Locating Acoustic Events Based on Large-Scale Sensor Networks

**DOI:** 10.3390/s91209925

**Published:** 2009-12-07

**Authors:** Yungeun Kim, Junho Ahn, Hojung Cha

**Affiliations:** Department of Computer Science, Yonsei University, Seodaemungu, Shinchondong, Seoul, Korea; E-Mails: ygkim@mobed.yonsei.ac.kr (Y.K.); junho@mobed.yonsei.ac.kr (J.A.)

**Keywords:** acoustic source localization, sensor network

## Abstract

Research on acoustic source localization is actively being conducted to enhance accuracy and coverage. However, the performance is inherently limited due to the use of expensive sensor nodes and inefficient communication methods. This paper proposes an acoustic source localization algorithm for a large area that uses low-cost sensor nodes. The proposed mechanism efficiently handles multiple acoustic sources by removing false-positive errors that arise from the different propagation ranges of radio and sound. Extensive outdoor experiments with real hardware validated that the proposed mechanism could localize four acoustic sources within a 3 m error in a 60 m by 60 m area, where conventional systems could hardly achieve similar performance.

## 1. Introduction

Acoustic source localization systems have been widely used in various applications such as counter-sniper systems [1-5], animal tracking systems [6,7] and a parking lot security system [8]. Although several systems have been developed to enhance accuracy, there are still restrictions with regards to the sensing range and an efficient acoustic source localization system that uses low-cost sensor nodes and works with minimum network overhead is still needed.

The sensing range of a conventional acoustic source localization system is restricted by the characteristics of the delivery ranges of sound sources and radio signals. If a sound is propagated more widely than the corresponding radio signal, the localization system may miscalculate because of difficulties in sharing information between sensor nodes. AML (Approximate-Maximum Likelihood) [9,10] and MBFM (Muzzle Blast Fusion Method) [1-5] have been proposed to solve this problem. AML uses the IEEE802.11 network, which has a broader range than IEEE802.15; however, the same problem occurs when the system covers a wider range than that of IEEE802.11. MBFM is a centralized system in which nodes, upon detecting an acoustic source, send all of the detected information to a base station; this generates network overhead since many nodes may concurrently send data to the base station.

The localization system [11,12] equipped with common sensor nodes is barely able to cover a wide area, due to the limited performance of the node hardware. If one acoustic source is propagated within multi-hop distance, the number of nodes that detect the source increases. This causes network overhead to share the detection information among the nodes. Resource-constrained nodes cannot completely handle the overhead; hence, packet losses increase. This may precipitate poor accuracy in the system. Moreover, the size of the shared data is often too large for the nodes to manage; thus, a system with low-cost nodes can usually sense only a limited area where communication occurs in single-hop fashion.

This paper proposes an acoustic source localization algorithm for systems with multi-hop communication, called MDSL (multi-hop distributed source localization). This process can localize multiple acoustic sources in a large area with low-cost sensor nodes and does not need additional hardware or a centralized communication process. In this algorithm, each node shares detected information within a one-hop range, and only one leader node selected in each one-hop group sends the result to the base node. At this point, miscalculated results arising from the limited communication within each group could be sent to the base node, but the base node can identify the inaccurate results and find an accurate position for the acoustic source. The system minimizes network overhead and detects multiple acoustic sources within a multi-hop range. Removing miscalculations can also be used to distinguish nodes that detect noise. The algorithm is implemented to operate with common sensor nodes, using low-cost acoustic sensors with no additional hardware. Extensive outdoor experiments with real hardware validate that MDSL can localize multiple acoustic sources in a large area with acceptable accuracy.

The paper is structured as follows: Section 2 discusses related work, followed by our research motivation in Section 3. The overall system architecture of the proposed mechanism is described in Section 4. The simulation and experiment results are given in Sections 5 and 6, respectively. In Section 7, we conclude the paper.

## 2. Related Work

Conventional acoustic source localization systems can be divided into DOA (Direction-of-Arrival)-based systems [13,14] and beam-forming-based systems [15-17]. DOA-based systems track an acoustic source by determining the direction of the acoustic source with an array of microphones on one sensor node. Therefore, DOA-based systems require powerful devices to handle a fast sampling rate, to distinguish the detection times of the microphones. The beam-forming-based systems track multiple acoustic sources by comparing the waveforms of the acoustic sources. These systems require even more powerful devices, such as PDAs, to perform complex algorithms with the significant amount of data generated.

More recently, several approaches have been studied: AML [9,10], MBFM [1-5] and DSL [11,12]. AML combines beam-forming and DOA to track multiple acoustic sources, but requires additional hardware based on the IEEE802.11 network; in addition, network overflow could occur if a target area is wider than the network range. MBFM checks the whole region where acoustic sources are likely to be produced. As low-cost sensor nodes cannot execute a high-complexity algorithm, every node must send detected information to the base station, which performs the calculation for final localization. This kind of centralized method [2-5] commonly produces network overhead, and the accuracy of the system is reduced.

DSL tracks a single acoustic source using low-performance sensor nodes. For any given acoustic source, the sensor nodes share their detection times, and the source location is determined by the node that detected the acoustic source the earliest. DSL performs poorly when an acoustic source is spread over a broad range. This is because sharing all of the detection information among nodes is difficult in practice in multi-hop networks. Another difficulty is the effective removal of miscalculated results caused by the characteristics of sound sources and radio signals. When multiple acoustic sources are simultaneously produced, it is even more difficult to locate the sources correctly.

To improve the shortcomings of these methods, this paper proposes a new algorithm that runs on inexpensive sensor nodes and detects single or multiple acoustic sources that simultaneously occur in a wide region.

## 3. Background

### 3.1. Distributed Acoustic Source Localization (DSL)

**Figure 1.**
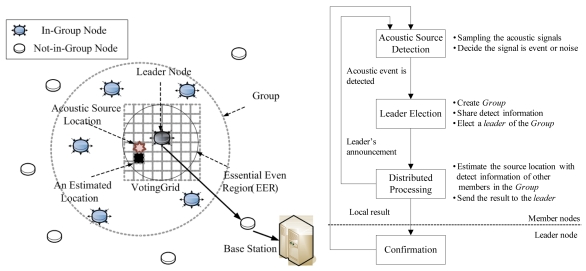
(a) DSL system overview (b) DSL algorithm overview.

The proposed system locates acoustic sources based on the DSL (Distributed Acoustic Source Localization) [11,12] algorithm. Figure 1(a) shows the concept of DSL for localizing an acoustic source. The group is constructed when an acoustic event occurs. In the group, one leader, the nearest to the source, is elected. All members of the group individually estimate the source location by exchanging the data of the acoustic source with each other. Finally, the leader node estimates the source location by gathering the results from other members. Because of this distributed mechanism, there is no computational overhead on the leader node in DSL. Figure 1(b) describes the detailed DSL localization process. The process is as follows:

- Acoustic Source Detection: When an acoustic event occurs, all of the nodes that detected the event construct the group exchanging information about their position and detection time.
- Leader Election: The node that has the earliest detection time is elected the leader node. The leader node constructs an *Essential Event Region (EER)* centered on itself. The *EER* is a simplified circular version of the Voronoi Region for resource-constrained WSN devices, and its diameter *W* is calculated as in Equation (1):

(1) $$W=\alpha \cdot \frac{\sum \left\|p_{l}-p_{i}\right\|}{h-1},i=1,2,\cdots ,h\;\text{and}\;i\neq l$$

Here, *^pi^* and *^pi^* are the locations of the leader node and *i*-th node in the group. *h* is the number of nodes in the group. is the coefficient for environmental constraints. The default value of α is 1, and manually decided by user to reflect the environment. The leader node constructs the Voting Grid by dividing the *EER* by a predefined size and announces it to the other members in the group.

- Distributed Processing: Each node individually estimates the source location by comparing its position and detection time with those of other members in the group. For each comparison, the partition line is defined as perpendicular to the midpoint of a line between two nodes. The source should be located on the side of the node that has an earlier detection time. Therefore, the right side grids of the Voting Grid score a point. After the comparison is completed, the voting result is sent to the leader node.
- Confirmation: The leader node aggregates the voting results from other members in the group and confirms the final source location.

**Figure 2.**
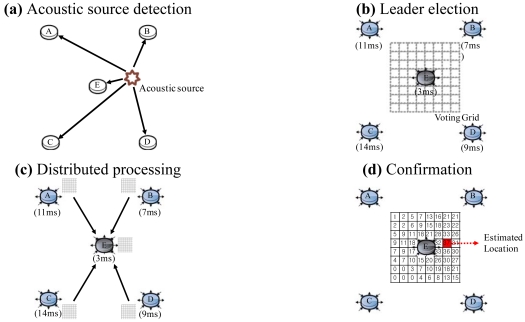
Example scenario of DSL.

Figure 2 shows an example scenario of the DSL algorithm. At first, node A, B, C, D and E detect an acoustic source as shown in Figure 2(a). They share their detection time and position. Node E, which has detected the source at 3 ms, is elected as a leader node (Figure 2(b)). Node E creates a Voting Grid based on its EER, and sends this to all other nodes. In Figure 2(c), each node votes on the Voting Grid by comparing the detection time difference and the distance with those of other nodes. The voting results are sent to node E, which aggregates the results from the nodes [Figure 2(d)]. The grid that has received the highest score of 37 is estimated as a location of the acoustic source.

### 3.2. Problems

DSL was previously evaluated in a small-scale environment, where all of the nodes were within a one-hop range. However, DSL may malfunction in large-scale environments, since an acoustic signal can propagate over a multi-hop range. We review the problems here, and then propose the MDSL algorithm as the solution, in the following section.

**Figure 3.**
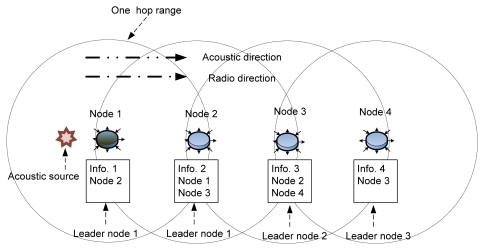
No virtual leader in a broad range.

DSL tracks an acoustic source by selecting the earliest node that detects the source as the leader node. When nodes are deployed in a large area and communicate in a multi-hop fashion, each node shares the detection data within a one-hop range; nodes outside this range cannot receive the data. One leader node is selected in every one-hop region where the source is detected, and several leaders may be generated, as detection may occur in more than one region. Consequently, localization could be wrongly conducted by these leaders with regard to a single acoustic source. However, this situation does not always happen when an acoustic source spreads over the multi-hop range. Figure 3 shows a case where the system reports only one result even though an acoustic source was very dispersed. Node 3 could choose node 2 as a leader, since node 2 detected a source earlier than node 3; however, node 2 could choose node 1 as a leader. Eventually, only node 1 is selected as the actual leader.

**Figure 4.**
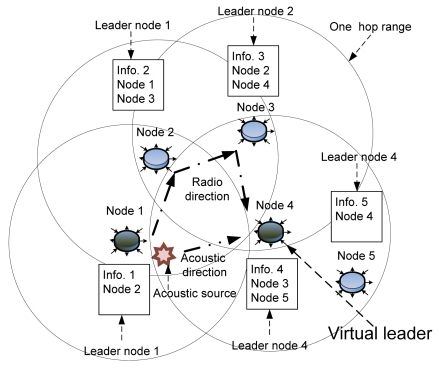
Virtual leaders in broad range.

There are two cases that generate wrong virtual leaders. One occurs when an acoustic sound is detected by more than two node groups, but these are too far apart from each other to communicate. The other occurs when the direction of the source is different from the direction of the data transmission, as shown in Figure 4. An acoustic source is produced near node 1. Node 4 can communicate directly with just one (node 3) of the nodes deployed within one-hop distance of node 1. Therefore, node 4 selects itself as the leader, since the node detects the source earlier than any other node in its group, thereby becoming a virtual leader, even though it is not the node nearest the acoustic source. If the flooding method is employed to overcome this situation, all nodes that detect the acoustic source should communicate to share detection information. However, the network overhead then increases and a large memory space is required to manage the shared data. Low-cost sensor nodes cannot handle these problems due to hardware limitations.

Other problems with DSL are the absence of management for detecting errors from the sensor nodes and for noise detection. The acoustic sensor in each node is low-performance hardware; hence, error sensing may occur frequently and randomly. The system would perform poorly if nodes that detect the noises were not appropriately separated.

## 4. Distributed Acoustic Source Localization in Large-Scale Sensor Networks

### 4.1. System Overview

**Figure 5.**
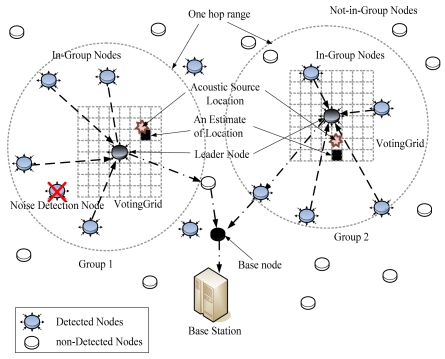
MDSL overview.

Figure 5 illustrates how MDSL tracks two acoustic sources produced simultaneously. Every node is equipped with one microphone, and the MDSL program is installed on each node. The detection time is synchronized to the global time. When acoustic sources are produced, every node that detects them transmits the detection time, along with its unique node ID and position, to other nodes within a one-hop range. The node that detects the source the earliest is selected as the leader, based on the collected data. Using the Essential Event Region created around the leader node, the range where the source could belong is calculated, and nodes that detected extraneous noises are removed. Localization is then performed using the DSL algorithm. Each node sends the detection data to the leader node, which sends its result to the base node. In certain cases, when an acoustic source is spread over a multi-hop range, virtual leader nodes are generated that are unable to perceive the real leader node. These virtual leader nodes also send their results to the base node. The base node can distinguish virtual leader nodes within the received results by analyzing the Essential Event Region. This part of the process is regarded as a centralized method, but virtual leader nodes are generated only in special cases; thus, network overhead rarely becomes a problem. We analyze the virtual leader overhead in Section 5.

### 4.2. Algorithm

MDSL improves on DSL by making it possible to detect multiple acoustic sources produced in regions where nodes communicate in multi-hop. The key idea of MDSL is that it separates the nodes that detected noise, and removes the miscalculated results during the localization step. MDSL can detect only one acoustic source within a one-hop range; in other words, we assume that acoustic sources produced simultaneously are in different one-hop regions. This is acceptable, since the system aims to cover a very large region. The simultaneous sources are defined as the sound events that are produced while nodes detect a source and that share their detected information with neighboring nodes. We use the PER (Possible Event Region) to achieve high accuracy in localization. The PER is easily calculated by expanding the concept of the EER in DSL.

#### 4.2.1. Possible Event Region

**Figure 6.**
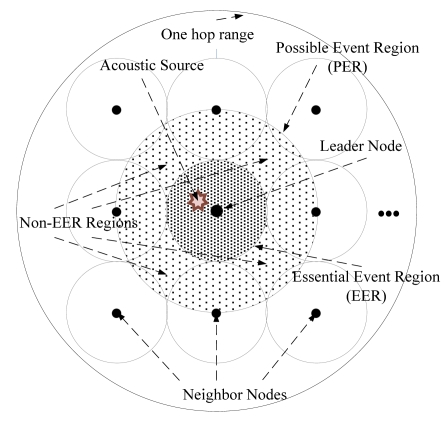
MDSL Possible Event Region.

Figure 6 illustrates that the *PER* is constructed by expanding the *EER* around the leader node. The *EER* is the region where an acoustic source was probably produced. Since the region has a circular shape to lower its computational complexity, a *non-EER* region is formed as shown in Figure 6. When an acoustic source is produced in this region, the system cannot appropriately track the source, since its accurate location is not included in the *EER*. Therefore, we replaced the *EER* with the *PER*, which is the circle including its *EER* and most of the *non-EER* in the vicinity. The radius *r_PER_* of the *PER* is obtained as in Equation (2):

(2) $$r_{\text{PER}}=|\left|p_{l}-p_{\text{far}}\right||-r_{\text{EER}}$$

where *p_l_* means the position of leader node, *p_far_* indicates the position of the farthest node from the leader node in the group, and *r_EER_* is the radius of the *EER* obtained from Equation (1), with a default α value. The *PER* is superior to the *EER* because the inaccuracy of localization, which is calculated from a source produced in the *non-EER*, is reduced. The computational overhead of the *PER* is also kept as low as that of the *EER* by finding the farthest node in the vicinity.

#### 4.2.2. Noise Removal

Sensor nodes are deployed in various environments; hence, these are likely to detect unexpected noises that consequently reduce localization accuracy. MDSL removes noises using the *PER*. Each node decides whether it detected the same source as a leader node by using the *PER*, the distance from the leader node and the difference in detection time. In other words, the time difference multiplied by the velocity of sound is calculated as a distance unit. The system removes noise by evaluating if it is included inside the *PER*, using the difference between the real distance among nodes and the calculated distance by detection times. We consider two extreme cases: an acoustic source that is produced exactly at the middle of two nodes or just at the leader node. In the former case, the calculated value is equal to the distance between the nodes. The calculated distance is 0 because the two nodes have the same detected time, due to simultaneous detection of the source. In the latter case, the calculated distance is the same as the real distance, because of the gap between the detected times. We divide the calculated distance by 2, since the sound that the leader node detects can be no closer than halfway between them. If the sound is produced in the *non-EER*, the value in the latter case exceeds the middle value, and the node is considered to have detected a noise. Therefore, noises are removed under the condition given in Equation (3) using the *PER*, instead of the *EER*, because a real acoustic source could be regarded as noise.

(3) $${\text{Dist}}_{\left(L,i\right)}<(i_{T}-L_{T})\nu \;\text{or}\;\frac{{\text{Dist}}_{\left(L,i\right)}-\left(i_{r}-L_{T}\right)\nu }{2}>{\text{PER}}_{L}$$

*Dist*_(_*_L,i_*_)_ represents the distance between a leader node and the *i*-th node. *i_T_* is the detection time of the *i*-th node, and *L_T_* is the time of leader node. represents the velocity of sound and *PER_L_* the radius of the leader node's *PER*.

**Figure 7.**
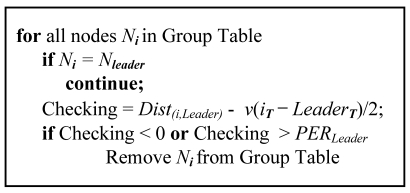
Pseudo code for noise removal.

Figure 7 shows the pseudo code that removes nodes that detected noise. First, nodes in the group receive the *PER* from the leader node. All nodes in the group are then checked to confirm whether the noise is included in the *PER* by Equation (3). If it is not, a noise is considered to have been detected, and the node is excluded from localization.

#### 4.2.3. Virtual Leader Removal

Virtual leader nodes are removed using the *PER* of the leader node. Each node that detects the acoustic source broadcasts the data to its one-hop neighbors. At this point, nodes that are unable to receive information about the real leader node select virtual leader nodes among them. Every leader node sends its localization result to the base node, regardless of whether the leader node is a virtual one. However, the virtual leader nodes are detected at the base node by the following method.

All of the leader nodes send information about their detection time and the *PER*. Since the real leader node is the closest to the source, the real leader node detects the signal earlier than the virtual leader nodes, so the virtual leader nodes are distinguished by comparing their detection times. Using Equation (4), a node that is included within the *PER* is chosen as a virtual leader node, and the others are considered real leader nodes that detected different acoustic sources from each other.

(4) $$0\leq \frac{{\text{Dist}}_{\left(i,f\right)}-(j_{T}-i_{T})\nu }{2}\leq {\text{PER}}_{i}$$

Here, *Dist*_(_*_i,j_*_)_ represents the distance between the *i*-th leader node and the *j*-th leader node. *i_T_* and *j_T_* are the detection times of the *i*-th and *j*-th leader nodes, respectively. is the velocity of sound, and *PER_i_* represents the radius of the *i*-th leader node's *PER*.

**Figure 8.**
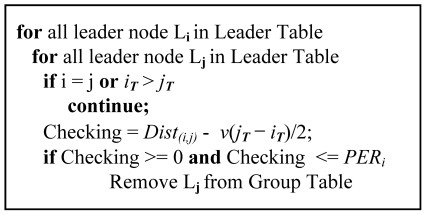
Pseudo code for virtual leader removal.

Figure 8 shows the pseudo code for removing virtual leader nodes. The base node stores collected data from leader nodes to the *Group Table*. Each set of data in *Group Table* is then compared to the others to check if its *PER* includes their position. If the *PER* includes one of them, the same source was detected, and it is excluded from localization.

## 5. Simulation

We conducted a simulation to evaluate the performance of MDSL when nodes are deployed in a large multi-hop area and multiple acoustic sources are simultaneously produced. Virtual leader nodes are generated if the sound spreads over the range of the radio signal. We simulated this situation and examined whether MDSL tracks the accurate position of the source by adequately removing virtual leader nodes.

The simulation was performed in a rectangular region where ten communication hops are needed in width and eight in height. One thousand nodes were randomly deployed in this region. The number of acoustic sources was from 1 to 7, and sources were produced in random positions and propagated over the whole region. With simulation, each node detected only one source that was the closest, because nodes begin calculations for the localization of their sources without detecting other sources. The detection data was shared with nodes within one-hop range, and the earliest node among each group was selected as the leader node.

**Figure 9.**
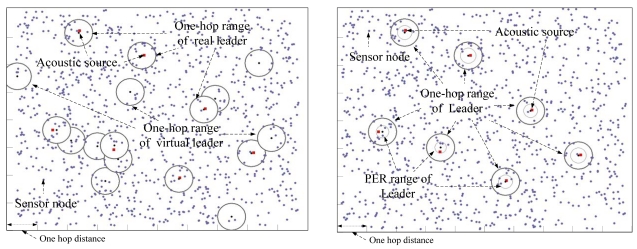
(a) Created virtual leaders when seven sources are produced. (b) The simulation result after the virtual leaders are removed by MDSL.

**Figure 10.**
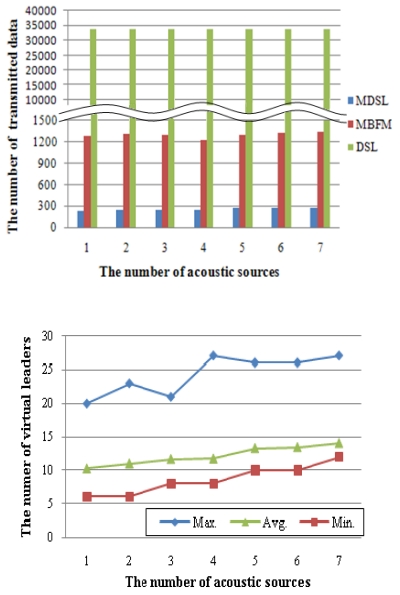
(a) Transmission overheads of MDSL, MBFM and DSL. (b) Number of minimum, average and maximum virtual leaders.

Virtual leader nodes were generated at this point. Figure 9(a) shows simulation results indicating that virtual leaders appeared when seven sources are produced. Nodes construct groups depending on the sources, and the number of groups is larger than the number of sources. Figure 9(b) shows results after removing virtual leader nodes from Figure 9(a), using the MDSL algorithm. The simulation was repeated independently 100 times according to the number of acoustic sources. The performance of MDSL is analyzed based on how many times the virtual leader nodes were removed. Figure 10(a) shows the transmission overhead of MDSL, MBFM and DSL in a large area, assuming error-free data transmission, as the number of acoustic sources increased. All nodes that detect acoustic sources in MBFM forward their detection data into a base server. The definition of the y axis is the number of transmissions in the case of sharing the detection information and routing the results from the leader nodes. We assume that the base server is located in a fixed position. Therefore, the number of transmissions in MDSL, MBFM and DSL are around 300, 1,300 and 33,000, respectively. Thus, the proposed mechanism is shown to efficiently manage network overhead that arises from radio transmission among multiple-hop nodes covering a large area.

Figure 10(b) shows the number of minimum, average, and maximum virtual leaders, depending on the number of acoustic sources. As the number of sources increases, the maximum number increases to 20, 23, 21, 27, 26, 26 and 27, respectively. Approximately 30 virtual nodes out of 1,000 are generated and participate in routing in the worst case. The proposed algorithm effectively reduces network overhead for localization. When one acoustic source is used, 10.24 virtual leader nodes are created on average. As the number of sources increase, the average number of virtual leader nodes increases, to 10.99, 11.65, 11.72, 13.39, 13.43 and 14.03, respectively. As more acoustic sources are produced, more virtual leader nodes are generated. This is because of the increased number of nodes that detect different sounds within the same group, on average. However, the number of virtual leader nodes is very small compared to that of all nodes.

**Figure 11.**
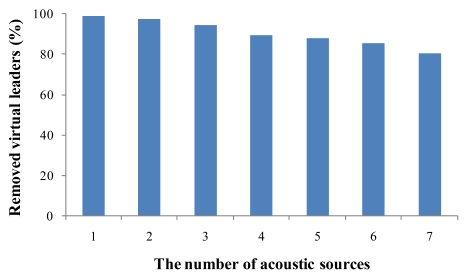
Virtual leaders removed by MDSL.

Figure 11 shows the percentage of nodes removed by the MDSL algorithm among the virtual leader nodes. In the case of one acoustic source, 99.02% of the virtual leader nodes are removed. As the number of acoustic sources increases, 97.18%, 94.59% 89.33%, 87.98%, 85.33% and 80.18% are removed, respectively. We see that the percentage of removal is higher than 80% in every case, although the percentage drops as the number of acoustic sources increases. We should note that almost all virtual leader nodes are removed in the simulation using only one producing source; in practice, multiple acoustic sources are rarely produced simultaneously.

**Figure 12.**
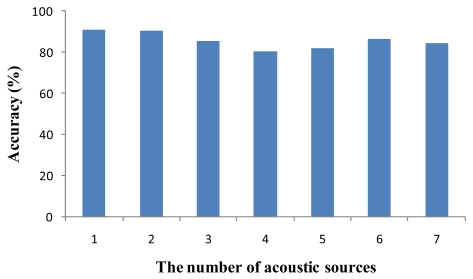
Localization accuracy of MDSL.

Figure 12 demonstrates the accuracy of MDSL. In this figure, the accuracy is represented by the percentage of a distance error with deployment size. For example, when the deployment size is 100 m × 100 m and the distance error is 1m, then the percentage accuracy is 99%. If multiple sounds are produced within a one-hop range, only one source is detected, and this influences the localization result. When one source produced sound, 91.06% accuracy was accomplished, and in cases where from 2 to 7 acoustic sources were active, the detection accuracy was 90.28%, 85.54%, 80.27%, 82.10%, 86.61% and 84.35%, respectively. MDSL consistently achieves high accuracy of more than 80% although the accuracy drops as the number of acoustic sources increases.

With this simulation, MDSL is shown to be effective for performing localization of multiple acoustic sources in a large area.

## 6. Experiments

### 6.1. Experimental Setup

**Figure 13.**
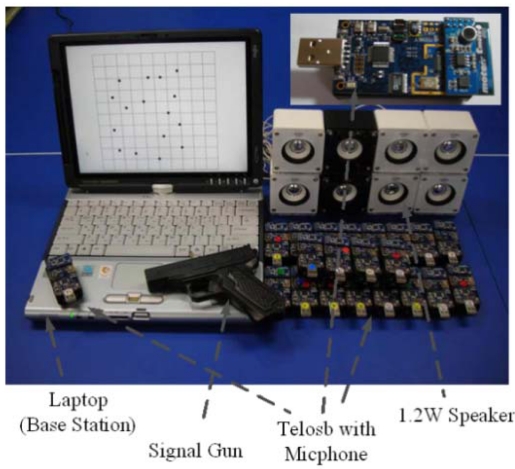
Experiment setup.

Figure 13 shows the devices used for the experiments. We used 16 Telos [18] motes, which were equipped with one microphone each, and we used four pairs of two 1.2W speakers to simultaneously produce multiple acoustic sources. One mote was configured as a base node and connected to a laptop via a serial port. The motes were running the RETOS operating system [19,20]. The maximum sampling rate of the microphone was 2.8 KHz in this setup. Using ETA [21], which employs a reactive method for time synchronization, our system maintained time synchronization while routing the time information. The experiments proceeded as follows: Section 6.2 discusses the detection time error and its solution in MDSL, followed by experiments on the localization accuracy and response time in Section 6.3. Finally, the experimental results in a multi-hop environment are given in Section 6.4.

### 6.2. Detection Time Error

**Figure 14.**
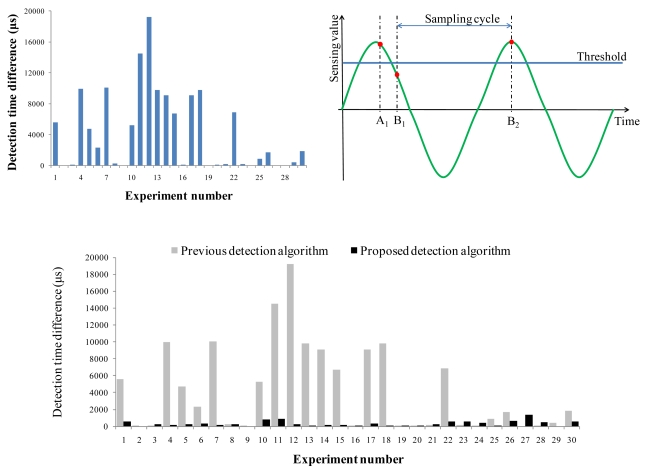
(a) Detection time error in MDSL. (b) Cause of the detection time error. (c) Reduction in detection time error with the proposed algorithm.

MDSL localizes an acoustic source based on the TDOA algorithm; hence, the accuracy of the detection time is a key factor determining the localization accuracy of MDSL. We conducted experiments to measure the detection time error in MDSL. Two nodes were deployed at the same position and an acoustic source was produced 5m from the nodes. We then measured the difference in detection times between the two nodes. As shown in Figure 14(a), the maximum difference was 19,600 μs in 30 tests, which is sufficiently large to induce a significant localization error. To understand the cause of this timing difference, we conducted further experiments. We first checked the time error that could have been caused by the ETA time synchronization algorithm. We placed two nodes at the same position, then turned on the LED of one node when its current time was sent to the other node, which subsequently turned on its LED upon receiving the packet. We compared the time difference of the two LED signals measured by an oscilloscope with that calculated by ETA. The experimental results showed that the detection time errors caused by ETA were lower than 30 μs in 30 tests, which was insignificant and would not affect the accuracy of MDSL.

Second, we analyzed the inherent error in detection time due to the low sampling rate of the sensor employed in MDSL. Figure 14(b) illustrates the cause of detection time error with the wave form of the acoustic source. A_1_, B_1_ and B_2_ indicate the sampling times of nodes A and B. At time A_1_, node A detects an acoustic event since the sensing value is higher than the threshold. Node B cannot detect the acoustic event at time B_1_ since the sensing value is lower than the threshold. Therefore, node B will detect an acoustic event at time B_2_. This detection time error on node B reduces the localization accuracy of DSL. To address this problem, we developed a detection algorithm, which makes use of two levels of thresholds and a buffering technique. Two thresholds are provided, where the second threshold is defined as half of the first one. In addition, a buffer is used to maintain the measured sensing value and the corresponding measurement time. When the sensing value is higher than the first threshold, the system looks for the buffer to find a more accurate detection time. The detection time is replaced by the corresponding measurement time of the sensing value, which is the first sensing value higher than the second threshold in the buffer. With this algorithm, the detection time of node B in Figure 14(b) is now replaced by B_1_. Figure 14(c) shows the result of the new detection algorithm in the same experiment. The time difference is 1,856 μs at most, and the average time difference is 338 μs, which is significantly smaller than the 4,297 μs obtained with DSL. This result shows that the proposed detection algorithm effectively reduced the detection time error caused by low sampling rate.

### 6.3. Localization Accuracy and Response Time

**Figure 15.**
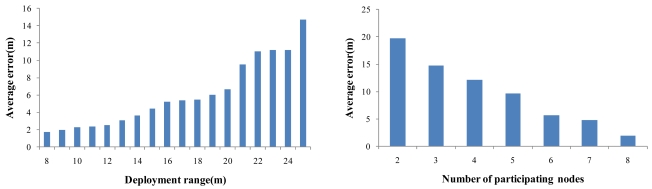

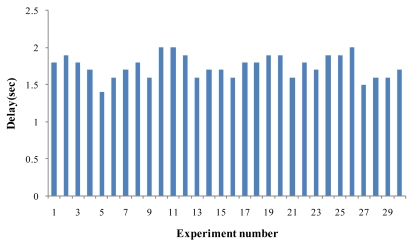
(a) Deployment range *vs.* localization accuracy when five nodes are deployed. (b) Localization accuracy when the deployment range is 20 m. (c) Response time of MDSL when eleven nodes are deployed in the basement of 90 m by 60 m with 20 m node spacing.

We conducted experiments to find appropriate node spacing for system deployment. We deployed five nodes with a node spacing that varied from 8 m to 25 m, and then measured the localization error ten times for each case. Figure 15(a) shows that the average error grew as the node spacing increased. In particular, the error was rapidly increased from 21 m node spacing and beyond. Based on this experiment, the node spacing of 20m was chosen for the subsequent experiments, as this value is considered reasonable in terms of both cost and accuracy. Further experiments were conducted to find the relation between the number of nodes and the localization accuracy. We localized an acoustic source in an outdoor space of 40 m by 20 m by changing the number of nodes from 1 to 8 with the same node spacing of 20 m. As shown in Figure 15(b), the distance error was 20m when only two nodes were deployed, while the error was lower than 2 m when seven nodes were used. This result shows that the localization accuracy of MDSL strongly depends on the number of nodes that detect the source. This is a tradeoff between the cost and the accuracy.

We additionally conducted experiments to estimate the response time of MDSL. Eleven nodes were deployed in a basement of 90 m by 60 m, with 20 m node spacing. We performed 30 measurements of the delay between the occurrence of an acoustic source and the completion of the localization process. As shown in Figure 15(c), the delay was less than 2 seconds, which was fast enough to respond to an acoustic event.

### 6.4. Results in a Multi-Hop Environment

We had three goals for evaluating the accuracy of MDSL in a large area where source localization is seemingly difficult to achieve. First, we analyzed how many virtual leaders were removed when one acoustic source was produced and the node groups were far from each other. We validated that our system tracked the acoustic source via the data gathered in the base node. Second, we measured the rate of removal of the virtual leaders when nodes were deployed randomly, without separate groups, so that virtual leaders were produced due to the different ranges between the sound and radio signals. We validated, through this experiment, that MDSL is an efficient system for localization with multi-hop communication. Finally, we measured the rate of removal of virtual leaders when multiple acoustic sources were simultaneously producing sound at a long distance from each other. Through this experiment, we validated that MDSL performs well even when multiple acoustic sources are active.

**Figure 16.**
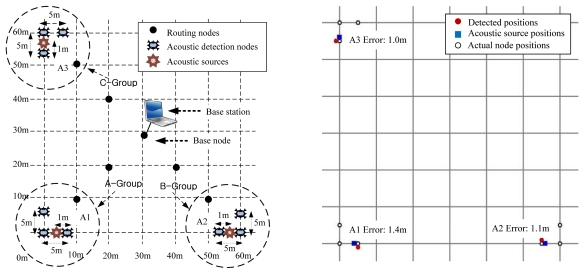
(a) Deployment among independent groups in broad range. (b) Result among independent groups in broad range.

First, we deployed nodes that were divided into separate groups and produced one acoustic source. We then measured the rate of removal of the virtual leader nodes. Figure 16(a) shows the deployment among independent groups, with three nodes in each group. We conducted this experiment in a large area (60 m × 60 m) to form separate groups, and each node was put on the ground to reduce the range of its radio signal. Nine nodes were used to detect an acoustic source, and three groups, each formed by three nodes, were constructed, which were designated ‘A’, ‘B’ and ‘C.’ Three acoustic sources 'A1′, 'A2′ and 'A3′ were produced, one by one, at different times by a signal gun. A1 and A2 were at a horizontal distance of 1 m from the closest node, and A3 was at a vertical distance of 1 m. Figure 16(b) shows the result of MDSL tracking for A1, A2 and A3. The distance errors were 1.4 m, 1.1 m and 1.0 m, respectively. When A1 was active, virtual leaders were generated in groups B and C; however, MDSL eliminated them and accepted only the leader node in group A. Virtual leaders were also created when A2 and A3 were produced, and MDSL also removed them. We validated that our system performed well when nodes were divided into separate groups.

**Figure 17.**
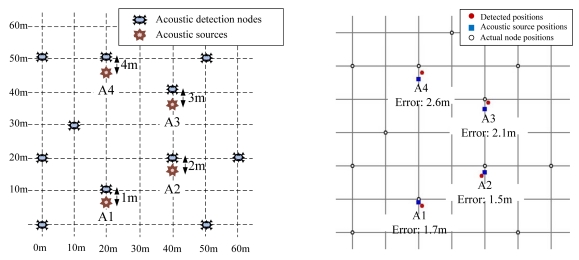
(a) Deployment for virtual leader removal in broad range. (b) Result in broad range.

Second, we deployed nodes evenly in a large area, and measured the accuracy of MDSL. Figure 17(a) shows the deployment of nodes and acoustic sources on the playground. We deployed 11 nodes in a large area of 60 m × 60 m, and each node was put on the ground to reduce the range of its radio signal. Four acoustic sources, designated ‘A1,’ ‘A2,’ ‘A3′ and 'A4′, were produced, one by one, at different times by a signal gun. Each source was produced at a vertical distance of 1 m, 2 m, 3 m, and 4 m from its closest node. Figure 17(b) shows the result of MDSL tracking for A1, A2, A3 and A4. The distance errors were 1.7 m, 1.5 m, 2.1 m and 2.6 m, respectively. These errors are acceptable, if we consider that the system is implemented with only low-cost sensor nodes in a large area. When A1 was produced, two virtual leaders were generated, and MDSL removed them. When A2, A3 and A4 were produced, 3, 3, and 2 virtual leaders were generated, respectively, and MDSL also removed all of these. With this result, MDSL was confirmed to be effective for tracking a single acoustic source with a number of nodes deployed in a large area.

**Figure 18.**
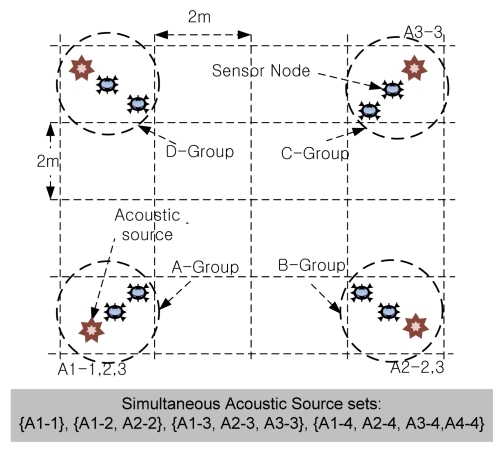
Deployment for MDSL virtual leader removal with increasing numbers of acoustic sources.

Finally, we measured the rate of removed virtual leaders when increasing numbers of acoustic sources were simultaneously producing sound at quite a long distance from each other. Figure 18 shows the deployment of the nodes and acoustic sources. We divided 8 nodes into 4 groups, designated ‘A,’ ‘B,’ ‘C’ and ‘D,’ so that each group consisted of 2 nodes to perform localization. The experiment was conducted in an area 8 m × 8 m square, and each group was deployed 2 m around its corner of this area. We recorded a clapping sound, which was produced by the speakers as an acoustic source. Each node communicated directly only within its group; in other words, the nodes of each group separated data received from others and only used the data from its group. This is because it is hard to perform an outdoor experiment using simultaneous acoustic sources, and the small experimental area allowed every node to communicate with the others. With this filtering, we obtained similar results to those that would have been obtained in multi-hop fashion. Since our speakers produced only unidirectional acoustic sources, we put speakers outside rather than inside the nodes. We performed the experiments by increasing the number of sources from 1 up to 4, in this order: {A1-1}, {A1-2, A2-2}, {A1-3, A2-3, A-3-3} and {A1-4, A2-4, A3-4, A4-4}, where the first number represents the position of an acoustic source and the second is the experiment number. We repeated each experiment 20 times.

In the experiment that used one acoustic source within group A, virtual leaders were generated in groups B, C and D; however, MDSL removed all of them. In the second experiment, with sound simultaneously produced by two acoustic sources within groups A and B, virtual leaders were created in groups C and D, and these were perfectly eliminated by MDSL. In the third experiment, a virtual leader was created in group D and removed by MDSL. In the last experiment, no virtual leader was generated when four acoustic sources were producing sound, and the system performed localization well. Thus, we were able to validate that MDSL is an effective system for tracking a number of simultaneous acoustic sources by removing the virtual leader nodes.

## 7. Conclusions

The contributions of this paper are three-fold. First, an efficient algorithm is proposed that allows localization to be performed in a large area that conventional systems can hardly cover due to the different ranges of acoustic sources and radio signals. Second, our system detects multiple acoustic sources that are simultaneously produced, enhancing its practicality when considering that multiple sources are sometimes generated in real-life environments. Finally, our algorithm has low enough complexity to be installed in low-cost sensor nodes. This is a very useful feature, allowing coverage of a large area with only low-cost sensor nodes. The experimental results validated that the proposed mechanism indeed detects up to four multiple acoustic sources that are produced simultaneously in a large area.
